# Carbogen gas-challenge blood oxygen level-dependent magnetic resonance imaging in hepatocellular carcinoma: Initial results

**DOI:** 10.3892/ol.2015.3526

**Published:** 2015-07-23

**Authors:** LONG JIANG ZHANG, ZHUOLI ZHANG, JIAN XU, NING JIN, SONG LUO, ANDREW C. LARSON, GUANG MING LU

**Affiliations:** 1 Department of Medical Imaging, Jinling Hospital, Medical School of Nanjing University, Nangjing, Jiangsu 210002, P.R. China; 2 Department of Electrical Engineering and Computer Science, Northwestern University, Evanston, IL 60611, USA; 3 Department of Biomedical Engineering, Northwestern University, Evanston, IL 60611, USA

**Keywords:** carbogen, blood oxygen level-dependent, magnetic resonance imaging, liver tumors

## Abstract

The present study aimed to evaluate the feasibility of performing carbogen gas-challenge blood oxygen level-dependent (BOLD) magnetic resonance imaging (MRI) measurements in patients with hepatocellular carcinoma (HCC). A total of 25 patients with HCC underwent T2* mapping derived from multi-echo gradient-recalled echo imaging prior to and following breathing carbogen (95% O_2_ and 5% CO_2_) for 10 min. Follow-up T2* mapping was performed in 5 patients 1 day after transarterial chemoembolization (TACE). T2*, R2* and ∆R2* values (R2*air - R2*carb) of the whole tumor, the solid region of the tumor and the adjacent liver parenchyma were measured and compared in the patients with HCC. The T2* value of the solid region of the tumor following carbogen breathing was higher than the value following room air breathing (P<0.05), and the R2* value of room air breathing was higher than that following carbogen breathing (P<0.05). ∆R2* values of the tumor and the adjacent liver parenchyma prior to and following carbogen breathing were 2.4±7.8, 8.1±14.7 and 2.0±11.0 sec^−1^, respectively. R2* values were significantly decreased in 2 cases 1 day after TACE (17.8 vs. −3.4 sec^−1^ and 10.2 vs. 2.4 sec^−1^). Overall, carbogen gas-challenge BOLD MRI measurements are feasible in clinical settings and may serve as a novel functional biomarker for monitoring the treatment efficacy of embolic therapies for HCC.

## Introduction

Blood oxygen level-dependent (BOLD) functional magnetic resonance imaging (MRI), acting via modulation of the T2*-weighted signal by changes in the ratio of blood paramagnetic deoxyhemoglobin to diamagnetic oxyhemoglobin, has been widely used to investigate the neural basis of various brain diseases for ~2 decades (1). In more recent years, this technique has been extended to evaluate tissue oxygenation in other organs, including the myocardium (2), kidney (3), breast (4) and prostate (5). In the liver, BOLD MRI has been used to investigate tissue oxygenation changes following the administration of a variety of physiological challenges, such as oxygen, carbon monoxide, carbogen, ethanol and glucose, in rats (6,7) and humans (8,9). Carbogen-challenge BOLD MRI was previously used to investigate tumor microvessel density (10) and to assess the early response of liver tumors to chemoembolization (11) in a rat hepatoma model. Recently, the feasibility of BOLD MRI studies in the human liver has been demonstrated in healthy volunteers and 1 patient with chronic liver disease in a fasting and a postprandial condition (8) or following oral ingestion of glucose (9). However, to the best of our knowledge, the potential of carbogen gas-challenge BOLD MRI to evaluate hepatic diseases, such as liver fibrosis and tumors, has not been investigated in the clinical setting. Additionally, no studies have been published on the feasibility of carbogen gas-challenge BOLD MRI in evaluating oxygenation changes of liver tumors, and in assessing therapeutic efficacy and predicting prognosis in patients with liver tumors. Therefore, the present study reports the preliminary results of BOLD MRI in assessing the oxygenation changes of liver tumors after inhaling carbogen, and in assessing therapeutic efficacy and predicting prognosis of transarterial chemoembolization (TACE) in patients with hepatocellular carcinoma (HCC).

## Patients and methods

### 

#### Patients

The present Health Insurance Portability and Accountability Act-compliant prospective study was approved by the Institutional Review Board of Jinling Hospital (Nanjing, China) and all patients provided written informed consent. A total of 25 patients of Jinlong Hospital (22 males and 3 females; median age, 52 years) with HCC were included in this study. In the 25 patients, HCC was diagnosed by liver resection specimen (n=1), liver biopsy (n=10) or using updated American Association for the Study of Liver Diseases criteria (n=14) (12).

#### MR protocol and image analysis

All scans were performed using a clinical 3T whole-body MR scanner (Magnetom Trio; Siemens Medical Solutions, Erlangen, Germany) equipped with eight receiver channels and a gradient system with a maximum gradient strength of 40 mT/m and a slew rate of 200 T/m/sec. For signal reception, a combination of spine coil and body matrix coil was used. All patients first underwent conventional T1- and T2-weighted imaging for selecting imaging slice of T2* mapping of the tumor. Two slices with maximal dimension of the tumor were chosen for the T2* mapping. Next, T2* mapping with multi-echo gradient-echo sequence with 9 echoes (3.4, 8.0, 12.7, 17.3, 21.9, 26.5, 31.1, 35.5 and 39.2 msec) was acquired at the end of respiration during the patient's breath hold prior to and following breathing carbogen (95% O_2_ and 5% CO_2_) for 10 min via a soft face-mask system (Meinuo Medical Equipment Co., Ltd., Shanghai, China). A continuous gas flow of 15 l/min was ensured for sufficient gas breathing. Other imaging parameters were as follows: Time of repetition, 100 msec; echo train length, 9; intersection spacing, 4.6 msec; flip angle, 30°; number of slices, 2; slice thickness, 3.0 mm; field of view, 27.5×40.0 cm; matrix, 132×192; number of averages, 1; and bandwidth, 350 Hz/voxel. The scanning time was 9 sec. Of 25 patients, 4 patients underwent follow-up T2* mapping 1 day after TACE, and 1 patient underwent follow-up T2* mapping one month after TACE. The slices for T2* mapping were chosen to be as similar as possible as those prior to TACE.

For all T2* mapping analyses, either pre-TACE data or post-TACE data, T2* and R2* (1/T2*, in sec^−1^, reflecting oxygenation in tissue) values of the whole tumor, the solid region of the tumor and the adjacent liver parenchyma were measured from T2* maps collected prior to and following carbogen breathing. For the whole tumor, two regions of interest (ROI), including the whole tumor, regardless of tumor necrosis, at the two slices of T2* maps were drawn manually; for the solid region of the tumor, two ROIs were placed in the solid region of the tumor with the marked T2* changes prior to and following carbogen breathing in two slices of the T2* maps. For the adjacent liver parenchyma to the tumor, three ROIs were chosen with the size of ~1 cm^2^ for each slice of the T2* maps; their average value was used for the final analysis. ∆R2* was calculated using the following formula: ∆R2* = R2*air - R2*carb, where R2*air is the R2* measurement acquired at steady-state room air multiple-gradient-echo MR imaging and R2*carb is the R2* measurement acquired at steady-state carbogen multiple-gradient-echo MR imaging (7).

#### TACE procedures

The TACE technique in this study has been widely used in clinical setting. Briefly, the right common femoral artery was punctured with an 18-gauge single-wall needle using the Seldinger technique. A 5-F vascular sheath was placed into the right common femoral artery. With ﬂuoroscopic guidance, a 4-F glide catheter (Shanghai Medical Equipment Co., Ltd., Shanghai, China) was advanced over the guide wire into the desired hepatic artery branch, depending on the tumor location. Chemoembolization drugs were mixed in a 2:1 ratio of epirubicin (40 mg; Haizheng Pharmaceutical Factory, Taizhou, Zhejiang, China), oxalipatin (200 mg; Qilu Pharmaceutical Factory, Jinan, China), fluorouracil (1,000 g; Shanghai Xudong Haipu Pharmacy Co., Ltd., Shanghai, China) and non-ionic contrast material (30 ml; Ultravist; Schering, Berlin, Germany) to iodized oil (15 ml; Shanghai Xudong Haipu Pharmacy Co., Ltd.), the dose of which depended on the tumor size. Subsequent to slow effusion of the chemoembolization drugs and gelatin sponge particles (1 mm^3^; Wuhan Jiuzhou Medical Equipment Co., Ltd., Wuhan, Hubei, China), complete embolization of the feeding hepatic artery was achieved. The TACE procedures were performed by an interventional radiologist with 20 years of experience.

#### Statistical analysis

Statistical analysis was performed with SPSS version 17.0 (SPSS Inc., Chicago, IL, USA). A paired t-test or independent sample Student's t-test was used to assess the difference in R2* and ∆R2* measurements in the whole tumor, the solid region of the tumor and the adjacent liver parenchyma prior to and following carbogen breathing. P<0.05 was used to indicate a statistically significant difference.

## Results

### 

#### R2* measurements for patients with liver tumors

**Figure 1.**
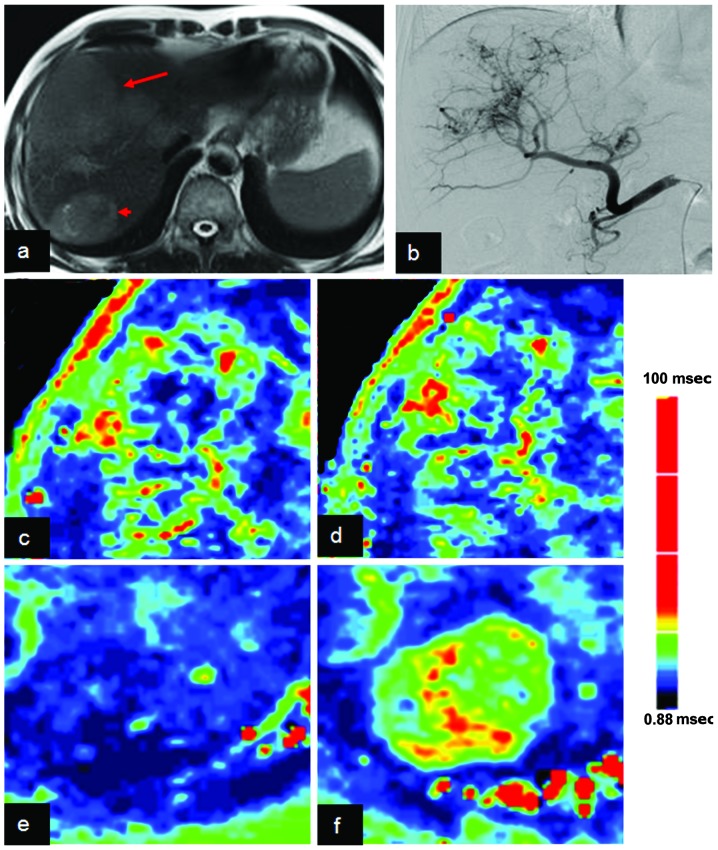
Gas-challenge T2* maps of HCC prior to transarterial chemoembolization in a 64-year-old male patient with diffuse hepatocellular carcinoma. (a) T2-weighted image of the liver showing multiple masses in the right posterior lobe (arrows). (b) Digital subtraction angiography showing the liver staining of the liver tumors. (c-f) Pre- and post-carbogen breathing T2* maps. Compared with T2* mapping (c and e) prior to carbogen breathing, the T2* value of the larger lesion is mildly increased (d and f) after inspiring carbogen gas; however, the small nodule exhibits a significant increase in T2* measurement following carbogen breathing.

**Figure 2.**
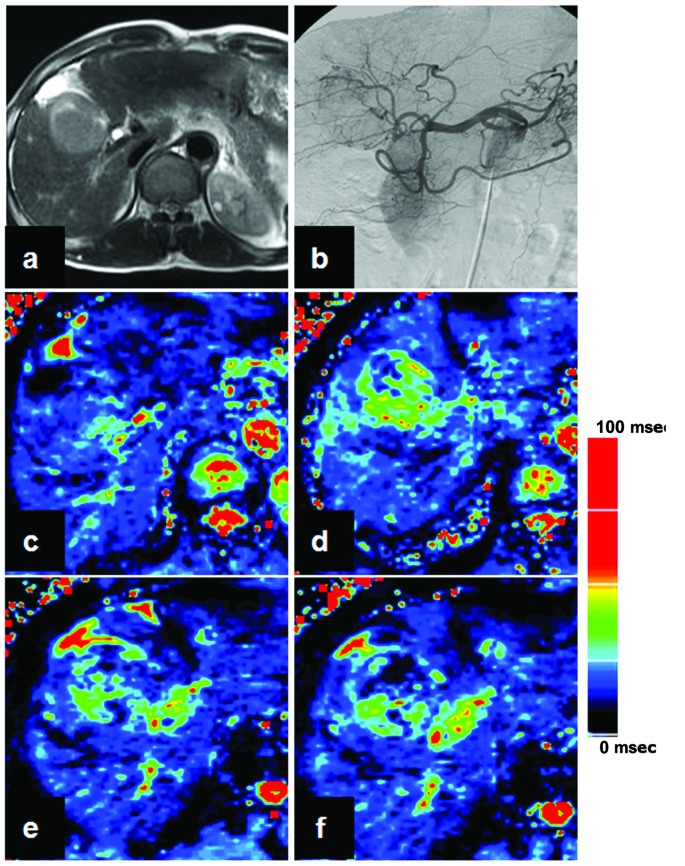
Gas-challenge T2* maps of hepatocellular carcinoma (HCC) prior to transarterial chemoembolization (TACE) and 1 day after TACE in a 60-year-old male patient with HCC. (a) T2-weighted image of the liver showing the high signal intensity round mass in the right anterior lobe. (b) Digital subtraction angiography showing the tumor staining of the liver tumor. Compared with T2* mapping (c) prior to carbogen breathing, the T2* value of the tumor is increased (d) after inspiring carbogen gas at the initial magnetic resonance evaluation. However, the TACE procedure leads to a marked reduction in the T2* value of the tumor (e) prior to and (f) following carbogen breathing.

A total of 25 patients with 25 tumor lesions on T2* mapping images were included into the final analysis. These tumors ranged in size from 2.3–18.2 cm. R2* (in sec^−1^) and ΔR2* (in sec^−1^) values of the whole tumor, the solid region of the tumor and the adjacent liver parenchyma prior to and following carbogen breathing are shown in Tables I and II. As shown in Table I, no significant difference was found in the R2* value of the adjacent liver parenchyma prior to carbogen breathing and following carbogen breathing (P>0.05). There was no difference in the R2* value of the whole tumor prior to and following carbogen breathing (P>0.05). For the solid region of the tumor, the R2* value of room air breathing was higher than that following carbogen breathing (P<0.05), which indicated increased oxygenation following carbogen breathing compared with prior to carbogen breathing. Following carbogen gas breathing, 2 patients exhibited marked T2* changes; however, the T2* changes were heterogeneous for these tumors with response to carbogen breathing. Representative cases are shown in Figs. 1 and 2. As shown in Table II, the R2*air and R2*carb values of the tumor were lower than those of the adjacent liver parenchyma (P<0.05). There was no significant difference in ∆R2* between the tumor and the adjacent liver parenchyma (P>0.05).

#### Tumor response

**Figure 3.**
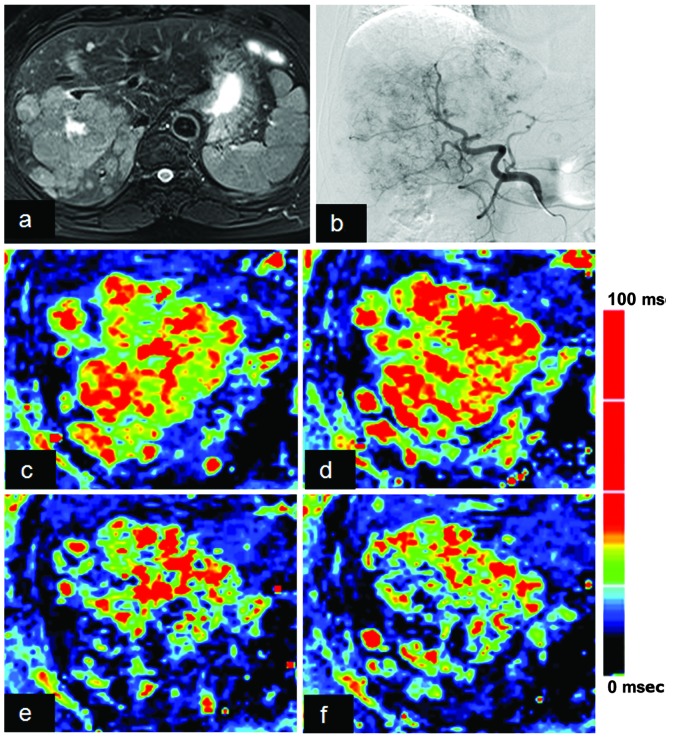
Gas-challenge T2* maps of hepatocellular carcinoma (HCC) prior to and 1 day after transarterial chemoembolization (TACE) in a 44-year-old male patient with HCC. (a) T2-weighted image with fat surppression of the liver showing the high signal intensity irregular mass in the right posterior lobe. (b) Digital subtraction angiography showing the tumor staining of the liver tumor. Compared with T2* mapping (c) prior to carbogen breathing, the T2* value of the tumor is increased (d) after inspiring carbogen gas at the initial magnetic resonance evaluation. However, the TACE procedure leads to a marked reduction in the T2* value of the tumor (e) prior to and (f) following carbogen breathing.

Of the 25 patients, 15 patients with HCC underwent TACE procedures. Carbogen gas-challenge BOLD MRI was performed in 4 patients with HCC at 1 day (n=4) and 1 patient at 1 month (n=1) post-TACE procedures; the ∆R2* value prior to TACE (∆R2* range, 0.5–17.8 sec^−1^ at the whole tumor for the 4 patients) was significantly lower than that following TACE (∆R2* range, −4.0 − 2.4 sec^−1^ at the whole tumor for the 5 examinations). A marked ∆R2* value was recorded in 2 patients at 1 day post-TACE (∆R2*, 10.2 vs. 2.4 sec^−1^ and 17.8 vs. −3.4 sec^−1^ at the whole tumor). A representative case is shown in Fig. 3.

## Discussion

This preliminary study demonstrates that carbogen-challenge BOLD MRI measurements are feasible in the clinical setting, and therefore have the potential to serve as a novel functional biomarker for monitoring the treatment efficacy of embolic therapies. To the best of our knowledge, this is the first study to focus on the liver tumor oxygenation changes in humans using carbogen-challenge BOLD MRI.

Of the physiological challenges used in BOLD MRI, carbogen (95% O_2_ and 5% CO_2_) is widely used in experimental and clinical studies. Carbogen administration is known to be safe in humans and has been clinically applied during radiation therapy in order to raise the oxygen tension and increase the radiosensitivity of the anoxic region. Compared with using pure oxygen, incorporating 5% CO_2_ is believed to counteract any oxygen-induced vasoconstriction (7). In the present human study, no subjects had any complaints with regard to the carbogen gas, indicating the safety of the gas in the clinical setting. In the study, 10-min carbogen gas breathing was performed rather than the block-designed scheme reported in our previous experimental study (7). In previous studies (7,8), block-designed BOLD MRI took a long time (>1 h) to measure the BOLD response in animals and humans, which limited the technique in clinical practice. The present preliminary study demonstrated the feasibility of a steady-state gas challenge to evaluate ∆R2* in human liver tumors. The T2* and R2* values of the tumor were found to be statistically different from those of the adjacent liver parenchyma. Haque *et al* (9) reported a statistically significant decrease in R2* (55.8±3.8 to 50.6±0.5 sec^−1^) following oral ingestion of 75 g glucose in 6 healthy subjects. Variable BOLD responses of HCC for carbogen breathing were observed in the present study; the reasons were complex, but included the fact that the tumors may have adapted to widely different perfusion environments. Additionally, variation in tumor microenvironments, including tumor vascularity, blood supply ratio of the hepatic artery to the portal vein and aerobic metabolic activity, can result in these variations of ∆R2* value (10). Thus, further studies are required.

The ~85% patients of HCC who are diagnosed at an advanced stage are not candidates for surgical therapy (13). In these patients, the established treatment protocol is TACE, which has proven survival benefits compared with best supportive care (14,15). Objective and accurate evaluation of the response of HCC to treatment is crucial for determining the requirement for repeated TACE or alternative treatment approaches, and for improving survival time (16). Multimodality MRI plays an increasingly important role in assessing the response of HCC to loco-regional therapy due to its lack of ionizing radiation, high contrast resolution and the possibility of performing functional imaging sequences (17). Dynamic contrast-enhanced MRI, diffusion-weighted imaging, perfusion-weighted imaging and MR spectroscopy have been used for the assessment of the early therapeutic response of large HCCs following TACE, each with their advantages and disadvantages (18). The present study presented a steady-state carbogen-gas challenge BOLD MRI method to monitor the response of HCC to treatment in humans. This steady-state carbogen-gas challenge BOLD MRI scheme is advantageous to perform in the clinical setting due to a shorter overall scan time compared with block designed BOLD MRI (10 min vs. >1 h) (8) and less contraindication compared with glucose challenge BOLD MRI (9). In the present study, it was found that TACE resulted in a decreased BOLD response of HCC compared with untreated HCC due to blocking of the tumor vessels. This indicated the potential of carbogen gas-challenge BOLD MRI as a biomarker to monitor treatment efficacy and predict the prognosis of TACE in patients with HCC. However, further studies in a large cohort are required to determine whether BOLD MRI has an ability to predict the prognosis of patients with HCC undergoing TACE. These preliminary results were supported by previously published experimental studies. For example, Rhee *et al* (19) showed that the use of 15-min oxygen-challenge BOLD MRI to monitor changes in hepatic tumor oxygenation following embolization was feasible in rabbits. Choi *et al* (11) found that carbogen gas-challenge BOLD MRI could monitor the early response of liver tumors to chemoembolization in a rat hepatoma model using a 9.4-Tesla scanner. The present findings have also been supported by a previous clinical study, which showed evidence for a correlation between tumor oxygenation and treatment outcome (18). Hypoxia is an important factor in radiotherapy treatment failure and, in clinical studies, has been associated with poor local tumor control and relapse in numerous cancer regions (18); a higher level of oxygen in the tumor tissue may improve the treatment response of these tumors (19,20).

However, the present study does have certain limitations. First, the study is only a preliminary report and the results are limited by a small heterogeneous sample size for liver tumor patients with variations in the size of the tumors and treatment options, which may have affected the statistical analysis of the study. Thus, a large-cohort study is required to further validate the value of carbogen gas-challenge BOLD MRI in predicting the liver tumor response to treatment in future. Second, respiratory motion during two T2* mapping acquisition prior to and following carbogen breathing can have an effect on T2* measurements, although as similar slices as possible were kept when imaging. Breathing instructions and a respiratory navigating or gating technique should be used carefully. Third, carbogen gas was arbitrarily used in this study when the optimal challenges remained underdetermined; further study is required to compare the BOLD responses during oxygen gas or glucose challenges with those during carbogen gas challenges. Fourth, T2* maps at two slices of maximal dimension of the tumor was acquired, which can confer bias for the evaluation of oxygenation changes of the whole tumor. However, encompassing the whole tumor would increase the scanning time and result in longer breath holding time, which would have decreased the image quality of the T2* maps. Last, the present study adopted a 10-min carbogen gas breathing scheme rather than a block-designed scheme, which was similar to that in task-related BOLD MRI in the brain. However, in our previous experimental study, the overall scanning time for block-designed BOLD MRI in the liver was too long to transfer to the clinical setting (7). The duration of carbogen gas breathing requires optimization in future studies.

In conclusion, the present preliminary study demonstrated that carbogen gas-challenge BOLD MRI measurements are feasible in the clinical setting. Carbogen-challenge BOLD MRI measurements have the potential to serve as a novel functional biomarker for monitoring the treatment efficacy of embolic therapies for HCC.

## Figures and Tables

**Table I.** Quantitative measurements of liver tissue and tumor prior to and following carbogen breathing.

Protocols	Whole liver tumor	Solid region of tumor	Adjacent liver tissue
R2*air, sec^−1^	44.7±15.3 (20.4–76.6)	39.2±19.6 (12.4–113.6)	102.6±37.1 (47.2–175.4)
R2*carb, sec^−1^	42.3±15.4 (19.3–81.3)	31.1±12.8 (9.9–54.1)	100.6±38.6 (42.6–166.7)
∆R2*, sec^−1^	2.4±7.8 (−17.5–19.8)	8.1±14.7 (−9.4–64.9)	2.0±11.0 (−19.6–22.0)

Data are expressed as the mean ± standard deviation (range). P>0.05 for R2*air and R2*carb of adjacent liver tissue, and R2*air and R2*carb of the whole tumor, according to paired t-test. P<0.05 for R2*air and R2*carb of the solid region of the tumor, according to paired t-test. R2*air, the R2*measurement acquired at steady-state room air multiple-gradient-echo magnetic resonance imaging; R2*carb, the R2*measurement acquired at steady-state room air multiple-gradient-echo magnetic resonance imaging; ∆R2*, R2*air - R2*carb.

**Table II.** Analysis of variance test for the quantitative measurements of liver tissue and tumor prior to and following carbogen breathing.

Protocols	Whole tumor vs. solid region of the tumor	Whole tumor vs. adjacent liver tissue	Solid region of tumor vs. adjacent liver tissue
R2*air, sec^−1^	0.441	<0.001	<0.001
R2*carb, sec^−1^	0.110	<0.001	<0.001
∆R2*, sec^−1^	-	-	-

-, unavailable data due to non-significance, according to analysis of variance test. R2*air, the R2*measurement acquired at steady-state room air multiple-gradient-echo MR imaging; R2*carb, the R2*measurement acquired at steady-state room air multiple-gradient-echo MR imaging; ∆R2*, R2*air - R2*carb.
